# Structure and properties of silk from the African wild silkmoth *Gonometa postica* reared indoors

**DOI:** 10.1093/jis/14.1.36

**Published:** 2014-01-01

**Authors:** Addis Teshome, S. K. Raina, Fritz Vollrath

**Affiliations:** 1 International Centre for Insect Physiology and Ecology, P.O. Box, 30772-00100, Nairobi, Kenya; 2 University of Oxford, Department of Zoology, Silk Research Group, South Parks Road, Oxford OX1 3PS, United Kingdom

**Keywords:** cocoon quality, scanning election microscopy, stress-strain curve, thermal property

## Abstract

African wild silkmoth, *Gonometa postica* Walker (Lepidoptera: Lasiocampidae), were reared indoors in order to examine the influence of rearing conditions on the structure and properties of silk cocoon shells and degummed fibers by using a scanning electron microscope, an Instron tensile tester, and a thermogravimetric analyzer. The cocoons reared indoors showed inferior quality in weight, length, width, and cocoon shell ratio compared to cocoons reared outdoors. There were no differences in cocoon shell and fiber surfaces and cross sectional structures. Cocoon shells were covered with calcium oxalate crystals with few visible fibers on their surface. Degummed fibers were smooth with minimum unfractured surfaces and globular to triangular cross sections. Indoor-reared cocoon shells had a significantly higher breaking strain, while the breaking stress was higher for cocoons reared outdoors. Fibers from indoor cocoons had a significantly higher breaking stress while outdoor fibers had higher breaking strain. Thermogravimetric analysis curves showed two main thermal reactions revealing the dehydration of water molecules and ir-reversible decomposition of the crystallites in both cocoons and fibers reared indoors and outdoors. Cocoon shells underwent additional peaks of decomposition with increased temperature. The total weight loss was higher for cocoon shells and degummed fibers from indoors. Rearing conditions (temperature and relative humidity), feeding method used, changes in total life span, days to molting, and spinning might have influenced the variation in the properties observed.The ecological and commercial significances of indoor rearing of G. *postica* are discussed.

## Introduction

Non-mulberry (wild) silk production is a unique eco-friendly agro practice for income generation and is compatible with conservation goals focusing on countering loss of forest biodiversity. Craig (2008) described “wild” silk as any type of silk other than that spun by the domesticated silkworm, *Bombyx mori* . Wild silk is produced all over the world by different species of silkmoths across a wide range of ecologies. About 80 silkmoth species in Asia and Africa are known to produce wild silk of economic value ( Jolly et al. 1975 ). Similarly, more than 60 species of wild silk producing insect species have also been identified in eastern Africa ( Kioko 1998 ). However, only four wild silkmoth species namely, *Gonometa postica* (Lepidoptera: Lasiocampidae), *Anaphe panda* , *Argema mimosae* , and *Epiphora bauhiniae* have been commercially exploited so far in different parts of Africa. Despite excellent properties and enormous economic potential, silk fibers from these silkmoths have largely escaped the notice of the modern commercial and scientific world until recently.

*Gonometa* species are polyphagous insects that feed on a number of acacia species and are known to produce high quality silk ( Ngoka et al. 2007 ; Gheysens et al. 2011). They are widely distributed in eastern and southern parts of Africa. Outdoor rearing predisposes the different life stages of the silkmoth to the vagaries of both biotic and abiotic conditions. Kioko (1998) and Ngoka (2003) observed *Mesocomys pulchriceps* and *Pediobus anasta-ti* as egg parasitoids, birds ( *Oriolus larvatus rolleti* , *Lanius collaris* and *Tockus albotermi-natus* ), and Formicidae ants ( *Camponotus spp* .,) as larval predators, and *Compilura spp* ., and *Coccygomimus spp* . as cocoon parasitoids of *G. postica* .

Several attempts have been made to reduce the early mortality of wild silkmoth larvae and pupae under field conditions. Semi-captive rearing of *G. postica* larvae in net sleeves was recommended to complement and augment the natural population ( Ngoka et al. 2007 ). Standardization of chawki (young silkworms) rearing of *Antherea mylitta* silkworms to the second moult stage to prevent early stage larval loss resulted in a 20% increase in the effective rate of rearing (Jayaparakash et al. 1993). The development of an artificial diet for *A. mylitta* containing *Asan* leaf powder also showed some success ( Akai et al. 1991 ). In addition to low productivity, sharp declines in biodiversity and population density of wild silkmoths are challenges for the sericulture industry ( Unni et al. 2009 ). In this regard, rearing wild silkmoths indoors could present a unique opportunity to conserve biodiversity through well-designed introduction and augmentative release programs to boost the natural population. However, such attempts can affect the level of production and the quality of cocoons ( Fening et al. 2008 ). In previous studies, cocoons of *A. mylitta* reared indoors had inferior quality to their outdoor counterparts (Shamitta and Rao 2006). So far, little or no information is available on efforts to cultivate African wild silkmoths for enhanced economic and conservation benefits. Thus, in this paper, we report the influence of indoor rearing on quality, structure, and properties of the cocoon shell and degummed fibers of *G. postica* .

## Materials and Methods

### Rearing

Total rearing of *G. postica* was undertaken from the brushing stage (immediately after hatching) to cocoon spinning in a rearing room at 22 ± 3ºC and 68 ± 7% RH. Live cocoons collected from Mwingi district, Kenya, were kept inside net sleeves until adult emergence. After mating, female moths were allowed to lay eggs on trays, and the eggs were kept under the same condition until hatching. Immediately after hatching, young worms were released on new leaves of acacia placed in horizontal wooden boxes lined with paper sheets, while older larvae were fed matured leaves. The rearing boxes were covered with plastic sheets and nylon net sleeves to avoid rapid desiccation of leaves and to prevent escape of the larvae. The paper sheets and remnant leaves and branches were changed every day to remove fecal pellets and maintain cleanliness in the rearing set-up except during periods of molting. Seven days after spinning, 30 male and 30 female cocoons were randomly selected and weighed with an electronic digital analytical balance with 0.1 mg readability. Cocoons were then cut with a sharp blade and were cleaned, and pupae and cocoon shells were weighed. Means were calculated with Proc *t* -test procedure (SAS 2010). Cocoon shell ratio was calculated as:

$CSR (\%) = [\text{weight of shell}/\text{weight of fresh cocoon shell}] \times 100$

Data on the physical properties of outdoor reared cocoons were obtained from Kioko (1998) for comparison.

### Scanning electon microscope study of cocoon shells and degummed fibers

Cleaned indoor and outdoor reared cocoon shells were boiled with 5 g/L of sodium carbonate for 90 min and were then soaked in star soft solution (a combination of two local-ly-available laundry and dish-washing detergents at a 1:1 ratio) of 50 mL/L of distilled water for 3 min and washed with hot and cold distilled water twice. Fibers were then placed in 70% ethanol for three days to remove remnant sericin and were dried at room temperature. Cross section slices of fibers were made by pulling them through an empty plastic tube, and a clean cut was made with a new razor blade. Randomly selected cocoon shells were pressed out into discs at the middle section with a sharp hole-punch for surface scanning electron microscope study. For cross sectional observation, cocoon shells were also cut into cross section slices using a sharp blade. Cocoon shell discs, degummed fibers, and cross sectional slices of fibers and cocoon shells were then mounted separately onto copper stubs using double sided tape and sputter-coated with gold for three minutes. The samples were then observed with scanning electron microscope (Jeol Neoscope, JCM-5000, www.nikon.com ) under an accelerating voltage of 10 kV with a beam current of 0.1 nA.

### Mechanical properties of cocoon shells and degummed fibers

An Instron 5542 instrument (500 N load cell, www.instron.com ) was used for tensile testing of cocoon shells. Five cocoon shells were used, and two strips (with dimensions of 10 x 15 mm) were cut from the middle sections of each cocoon shell. A tensile test was carried out at room temperature with a gauge length of 5 mm and a cross head speed of 2 mm/min ( Fujia et al. 2010 ). Samples of degummed fibers were prepared by gently pulling and cutting approximately 40 mm of fibers from the floss. Fibers were then mounted and taped across 10 mm long, rectangular cardboard, which was then fixed in the Instron instrument (5 N load cell). Before being tested, each specimen was examined under an optical microscope to ensure that only single fibers were used. Tests were conducted with a gauge length of 10 mm at a rate of 0.1 mm/sec and at 22ºC and 55% RH. Ten tests were made for each specimen to generate the average tensile stress-strain curve. Cross-sectional areas of to 400 cm ^-1^ with an average of 64 scans. All the fibers were calculated from the digital im-spectral operations were performed using ages of transverse sections on scanning Omnic 7.3 (Thermo Scientific). electron microscope micrographs and analyzed with ImageJ 1.42q Results ( http://imagej.nih.gov/ ). Normalized cross-sectional areas were obtained by averaging 50 Cocoon quality individual silk filaments for each sample. The Table 1 summarizes the physical properties of tensile parameters were calculated with a cocoon shells from male and female *G. posti-* home-designed program in Microsoft Excel *ca* reared indoors and outdoors (values in the (Tensile Import v. 2.0, www.microsoft.com ). parenthesis represent cocoons reared outdoors Stress-strain curves were plotted using Origin (data from Kioko 1998 )). There was a highly Pro 8 ( www.originlab.com ).

**Table 1.**
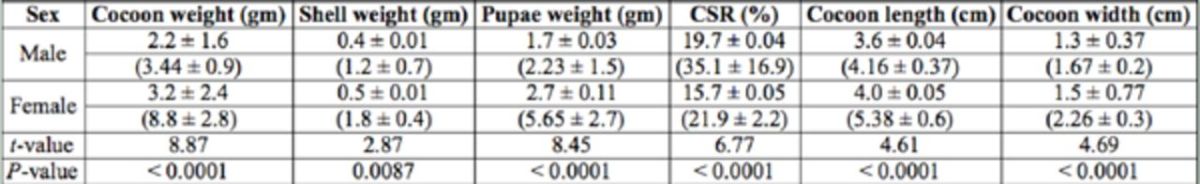
Mean ± SE of cocoon shell quality parameters of *Gonometa postica* reared indoors and outdoors. Values in parenthesis are measurements for outdoor cocoons from Kioko (1998) .

### Thermal degradation behavior of cocoon shells and degummed fibers

Cocoon shell discs (7 mm in diameter) were width and pupae weight, while males had a pressed out of the middle section of the co-significantly higher cocoon shell ratio. How-coon shells with a sharp hole-punch. ever, cocoon shells reared indoors were Degummed fibers were compressed and inferior in quality compared to those reared pressed out similarly. Thermogravimetric outdoors. The difference was even prominent analysis was performed using TA instrument in female cocoon weight and shell ratio. model Q500 ( www.tainstruments.com ). Three Males reared indoors had comparable cocoon tests were made for each sample. Temperature properties except for cocoon shell ratio, which ranges of 25–900 and 25–800°C for cocoon was notably lower. However, male pupae shells and degummed fibers, respectively, at a reared indoors were heavier than pupae reared heating rate of 20°C/min, an N2 flow of 60 outdoors. mL/min, and an air cool time of 40 min were used.

### FTIR study

A Nicolet 6700 FT-IR (Thermo Scientific, www.thermoscientific.com ) equipped with a liquid nitrogen cooled MCT-A detector was used. Three cocoon discs were sampled three times on the outside of the cocoon shell disk, and all the spectra were averaged. Spectrawere obtained at 4 cm-1 resolution from 7000 to 400 cm-1 with an average of 64 scans. All spectral operations were performed using Omnic 7.3 (Thermo Scientific).

## Results

### Cocoon quality

Table 1 summarizes the physical properties of cocoon shells from male and female *G. postica* reared indoors and outdoors (values in the parenthesis represent cocoons reared outdoors (data from Kioko 1998 )). There was a highly significant variation in cocoon shells for most of the parameters considered between males and females reared indoors. Females had significantly higher cocoon weight, length, and width and pupae weight, while males had a significantly higher cocoon shell ratio. However, cocoon shells reared indoors were inferior in quality compared to those reared outdoors. The difference was even prominent in female cocoon weight and shell ratio. Males reared indoors had comparable cocoon properties except for cocoon shell ratio, which was notably lower. However, male pupae reared indoors were heavier than pupae reared outdoors.

### Cocoon and fiber structures

**Figure 1.**
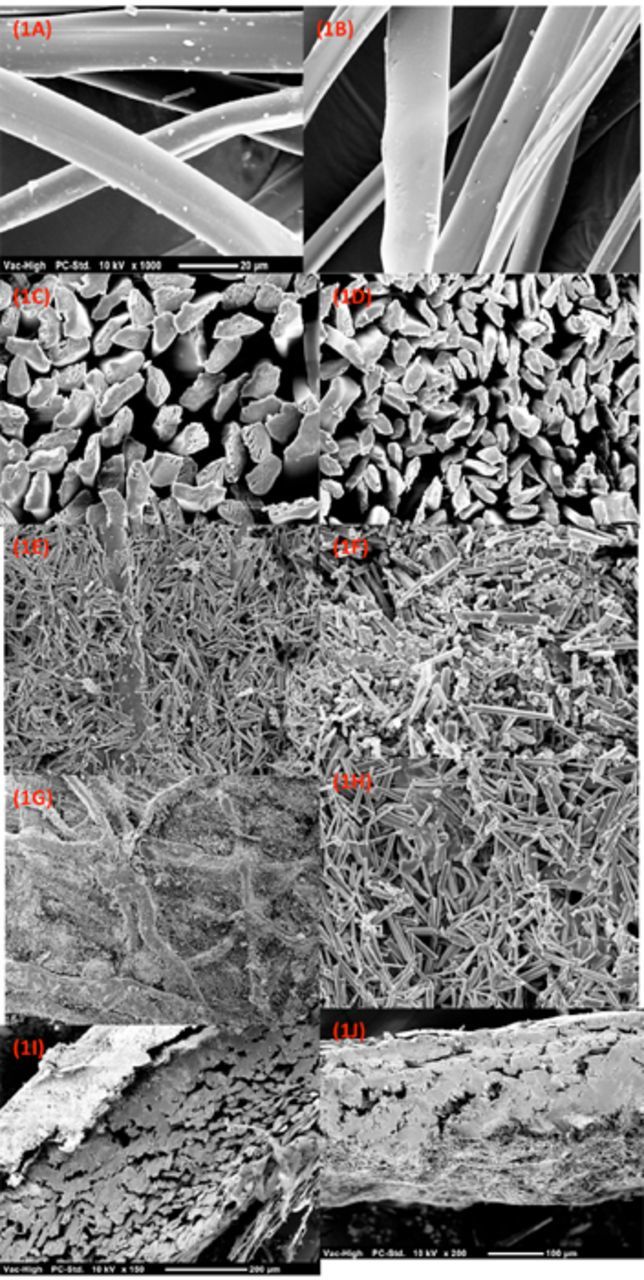
1A) Indoor fiber surface, (1B) outdoor fiber surface, (1C) outdoor fiber cross section, (1D) indoor fiber cross section, (1E) indoor cocoon shell surface, (6F) outdoor cocoon shell surface, (1G) outdoor crystals, (1H) indoor crystals, (1I) outdoor cocoon shell cross section, (1J) indoor cocoon shell cross section. High quality figures are available online.

**Figure 2.**
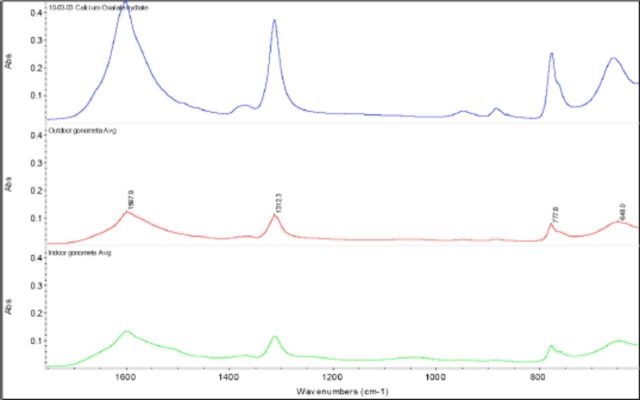
FTIR spectra of pure calcium oxalate (blue), outdoor-reared (red) and indoor-reared *G. postica* (green) outer cocoon shell surface. High quality figures are available online.

Figure 1 shows the surface and cross sections FTIR study of silk cocoon shells and degummed fibers of A Nicolet 6700 FT-IR (Thermo Scientific, *G. postica* reared indoors and outdoors. There www.thermoscientific.com ) equipped with a was no clear difference in the surface liquid nitrogen cooled MCT-A detector was structures and cross sectional shapes of used. Three cocoon discs were sampled three degummed fibers from cocoons reared indoors times on the outside of the cocoon shell disk, and outdoors ( Figure 1 A–D). There were and all the spectra were averaged. Spectra some artifacts of the degumming process and were obtained at 4 cm ^-1^ resolution from 7000 the spinning behavior, causing inconsistency in the diameter along the fiber axis. The average diameter of the fibers was smaller (16 µm) in indoor cocoons compared to outdoor cocoons (21 µm). The fibers had regular and unfractured surfaces with globular to triangular cross sections ( Figure 1 C, D). The surfaces of both cocoon shells were rough, covered with randomly arranged fibers, and showed countless cross bindings, wrinkles, and networking of twisted filaments ( Figure 1 E, F). The cocoons were multilayered and porous, with high sericin/gum content. Cocoon thickness was 0.536 mm for outdoor cocoons, which was considerably higher than indoor cocoons (0.326 mm). The cocoon shells were covered with crystals, though the crystals on the outdoor cocoons were larger in size than indoor cocoons ( Figure 1 G, H). The crystal layer was also thinner in indoor cocoon shells ( Figure 1 I, J). The FTIR spectra showed the peaks (at 1312/cm and 777/cm) were attributed to calcium oxalate monohydrate crystals (Sigma-Aldrich, www.sigmaaldrich.com ) ( Figure 2 ).

### Mechanical properties

**Figure 3.**
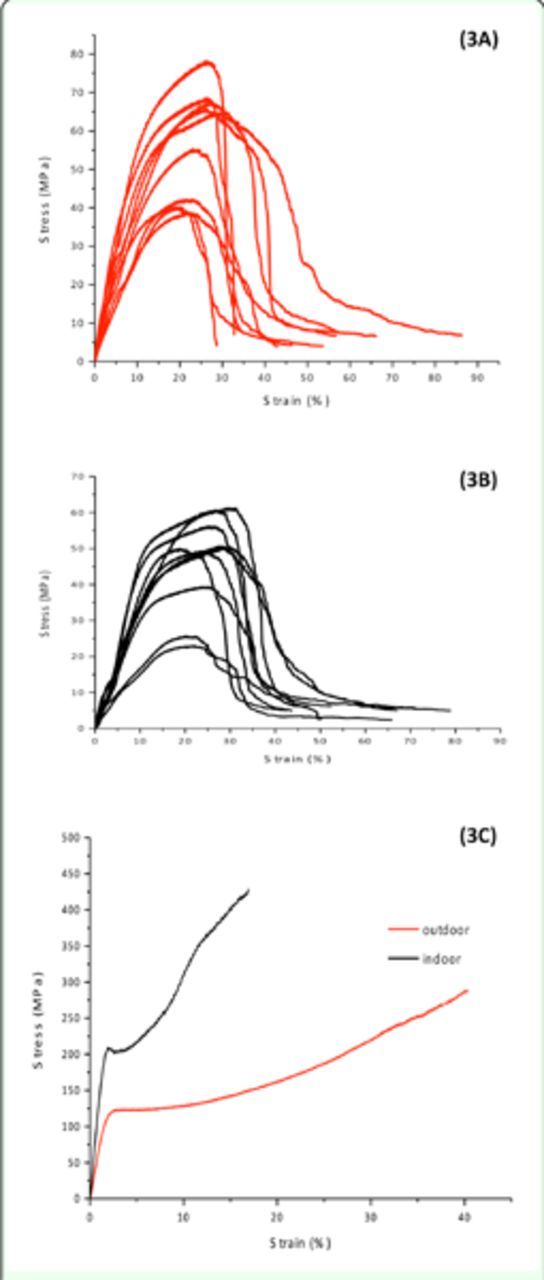
Stress-strain curves of *Gonometa postica* (3A) indoor cocoon shells, (3B) outdoor cocoon shells, and (3C) degummed fibers. High quality figures are available online.

The mechanical properties of cocoon shells and degummed fibers from *G. postica* reared indoors and outdoors are summarized in Table 2. There was a highly significant difference between the tensile properties of degummed fibers from indoor and outdoor cocoons. Indoor reared degummed fibers had higher initial modulus and breaking stress (tensile strength). Degummed fibers from cocoons reared outdoors had higher breaking strain and breaking energy. However, indoor and outdoor cocoon shells showed no significant dif-difference except for breaking energy ( Figure 3 ). Outdoor cocoon shells showed a signifi-hardening regions ( Figure 3 C). In contrast, in the cocoon shells, the binding points between fibers were observed to break gradually, and there was a rapid fall in stress, which indicates fiber bonding in the cocoon shells was broken and simple intertwined fibers were remained ( Figure 3 A, B).

### Thermal degradation behaviour

**Figure 4.**
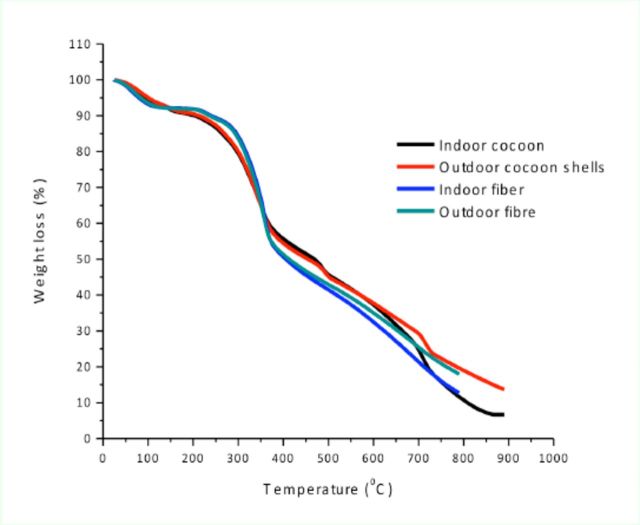
Thermogravimetric analysis curves for indoor- and outdoor-reared *Gonometa postica* silk cocoon shells and degummed fibers. High quality figures are available online.

Thermogravimetric analysis curves showed that there were no differences in the shapes of the graphs between indoor and outdoor degummed fibers and cocoon shells ( Figure 4 ). However, differences were observed between cocoon shells and degummed fibers. Cocoon shells showed steady weight loss due to dehydration while degummed fibers had rapid dehydration followed by a period of constant weight. Cocoon shells required a higher temperature for dehydration. The weight loss due to dehydration was 12.92% and 12.99% for indoor and outdoor degummed fibers, respectively. A substantial weight loss followed the gradual decrease in weight as heating temperature increased. Degummed fibers commenced decomposition at higher temperatures. Rapid weight loss commenced at 283 and 279°C for indoor and outdoor degummed fibers, respectively. However, the rapid degradation of indoor and outdoor cocoon shells started at 246 and 253°C, respectively. Unlike the degummed fibers, cocoon shells exhibited multistep de-compositions at 483–506 and 710–730°C. The total weight losses were 87.3% and 82.1% for indoor and outdoor degummed fibers respectively, and 93.4% and 86.4% for indoor and outdoor cocoon shells, respectively.

## Discussion

The results obtained demonstrate that rearing conditions influenced the quality and properties of silk produced by *G. postica* . The most significant cocoon commercial properties (size and weight) for *G. postica* reared indoors were inferior to those reared outdoors. Similarly, Shamitha and Rao (2006) found that cocoons of tasar silkworms reared outdoors also showed superiority in length, thickness, and weight of cocoon shell and peduncle over cocoons reared indoors. In contrast to our study, which showed differences in diameters of fibers, filaments of tasar silk in Shamith and Rao (2006) showed uniform diameter in both methods of rearing. *G. postica* reared inside took lesser days to complete their life cycle, and were smaller in size and weight (personal observations). This result, coupled with the feeding position, might have influenced the total amount of silk materials (sericin and fibroin) produced by the larvae, leading to substandard cocoon quality. Conventional indoor rearing of *Antheraea assamensis* by horizontal tray-feeding posture is associated with low food consumption and abnormal microvilli in the form of irregular arrangements, discontinuity, and less thickness than that of normal worms ( Sudip et al. 2009 ).

Unlike cocoon physical properties, cocoon shells and degummed fibers from indoor cocoons showed advantages in mechanical properties. Gheysens et al. (2011) reported 401 MPa and 443 MPa breaking stress for sodium carbonate degummed and demineralized (process of removing the calcium oxalate crystals) and pineapple juice degummed *G. postica* fibers, respectively, which are comparable to the present findings for degummed fibers from cocoons reared indoors (432.9 MPa). The results obtained for the cocoon shells were similar to those reported for *B. mori* cocoon shells by Zhao et al. (2005) , who reported a 25.4% ultimate tensile strain for *B. mori* complete cocoon shell. Huang et al. (2008) also reported a 54 MPa tensile strength for the normal compact *B. mori* cocoon shell. *B. mori* cocoon shells and fibers followed similar trends in weight loss when exposed to a temperature range of 105–550°C ( Zhang et al. 2002 ). These results for *B. mori* are in contrast with *G. postica* cocoon shells and fibers, which exhibited variability in their response to heat treatment. Degummed fibers had a higher decomposition temperature (283–286°C) and took fewer steps of decomposition than cocoon shells. The multistep decomposition for cocoon shells can be explained by the presence of a high concentration of calcium oxalate crystals on the surface. Outdoor cocoon shells had higher decomposition onset temperature, which indicate their contribution as self-thermoregulating structures. *G. postica* also showed a higher temperature for weight loss due to decomposition than muga and eri silk fibers (249°C) ( Bora et al. 1993 ).

Though current findings were not encouraging in terms of commercial quality parameters, they did show that *G. postica* is not averse to indoor rearing. With further fine-tuning of the present rearing method, it may be possible to develop more flexible, adoptable, and eco-nomically-viable methods that do not compromise quality and properties of the silk. Indoor rearing still can be utilized as a grain-age activity (seed production) integrated with well-designed augmentative release programs for enhancing and maintaining the natural population at an economically-profitable level. The excellent mechanical and high heat stability properties exhibited by silk fibers from *G. postica* reared indoors will present new opportunities for its further utilization. Indoor rearing can be a suitable intervention for maintaining equilibrium among the quick-ly-changing human demands, biodiversity conservation, and sustainable utilization of forest-based resources. However, care must be taken to avoid extinction of the wild population due to adaptation to conditions in captivity.
